# Humans use underestimates of auditory spatial and temporal uncertainty for perceptual inference

**DOI:** 10.1098/rspb.2024.2880

**Published:** 2025-06-04

**Authors:** Fangfang Hong, Jiaming Xu, Michael S. Landy, Stephanie Badde

**Affiliations:** ^1^ Department of Psychology, University of Pennsylvania, Philadelphia, PA, USA; ^2^ Department of Psychology, The University of Texas at Austin, Austin, TX, USA; ^3^ Department of Psychology and Center for Neural Science, New York University, NY, USA; ^4^ Department of Psychology, Tufts University, Medford, MA, USA

**Keywords:** Bayesian causal inference, multisensory integration, audiovisual spatiotemporal integration, sensory uncertainty

## Abstract

Making decisions based on noisy sensory information is a crucial function of the brain. Various decisions take each sensory signal’s uncertainty into account. Here, we investigated whether perceptual inferences rely on accurate estimates of sensory uncertainty. Participants completed a set of auditory, visual, and audiovisual spatial as well as temporal tasks. We fitted Bayesian observer models of each task to every participant’s full dataset. Crucially, in some model variants, the uncertainty estimates employed for perceptual inferences were independent of the actual uncertainty associated with the sensory signals. Model comparisons and analysis of the best-fitting parameters revealed that, in unimodal and bimodal contexts, participants’ perceptual decisions relied on underestimates of auditory spatial and audiovisual temporal uncertainty. These findings challenge the ubiquitous assumption that human behaviour optimally accounts for sensory uncertainty regardless of sensory domain.

## 1. Introduction

**Figure 1.**
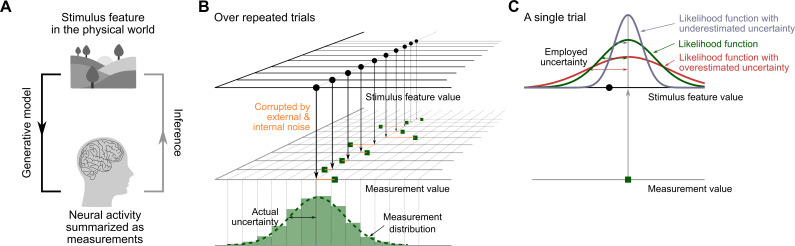
Generative model and perceptual inference. (A) An event in the world leads to neural activity that represents stimulus properties such as the stimulus location. The brain only has access to the measurement and has to infer the stimulus property that gave rise to it. (B) Due to external and internal noise, the measurements will not be perfectly aligned with the stimulus property in the physical world. The standard deviation of the measurements over repeated presentations of the same stimulus describes the observer’s actual sensory uncertainty. (C) In a single trial, the brain infers the physical property of the stimulus based on a single measurement; perceptual inference relies on the likelihood of each possible value of the stimulus property given both the measurement and its estimated uncertainty. For a symmetric measurement distribution, the shape of the likelihood function equals that of the measurement distribution if the uncertainty is independent of the true stimulus value.

Our senses are inherently noisy [1]; repeated exposure to the same physical stimulus rarely leads to the same neural activity in the brain and, thus, rarely to the same sensory measurement of the stimulus properties (figure 1A,B). Imagine your dog breaks free from its leash while you are in the woods. You hear a series of barks. Due to sensory noise, the sensory measurements of the barks’ locations vary, even if the dog stays in the same place. The probability distribution of the spatial measurements across many barks (the ‘measurement distribution’) reflects auditory spatial uncertainty; the broader the distribution, the higher the level of uncertainty. Consequently, a single bark might have originated from a range of plausible locations, each with its own likelihood of being the true origin (figure 1C). If decisions take sensory uncertainty into account, i.e. consider all possible locations and their likelihoods (the ‘likelihood function’), changes in sensory uncertainty should lead to specific changes in behaviour. Indeed, the degree of uncertainty associated with sensory signals influences our perception [2], cognition [3], action [4,5], memory [6], learning and decision-making [7,8], indicating that a wide range of human behaviours takes sensory uncertainty into account [9].

The match between sensory uncertainty estimates underlying perception and actual uncertainty remains contested. Bayesian encoding theory posits that uncertainty is automatically encoded in the activity of neurons selective for a stimulus feature. According to this account, the likelihood can be inferred from a probabilistic population code [10–13], implying that uncertainty is accurately represented and employed to mediate various decisions. This theory is supported by neurophysiological evidence showing that the likelihood function can be decoded from population activity in the primary visual cortex. Monkey behaviour in an orientation-classification task is better predicted by models using trial-specific estimates of uncertainty from neural responses, compared to a model that only derives a point estimate of orientation, suggesting that trial-specific likelihood functions underlie perceptual decisions [14]. In humans, likelihood functions have been decoded from fMRI measurements of visual cortical activity and linked to performance in visual orientation estimation [15] and spatial working memory tasks [16]. Few psychophysical studies provide direct evidence that internal likelihood functions accurately mirror sensory uncertainty (figure 1B,C). These studies contrast models with accurate or inaccurate likelihood functions for a visual-spatial localization task [17] and a visual orientation change-detection task [18]. They find that uncertainty is accurately encoded as a likelihood function. These studies focus on visual location or orientation, features known to be encoded by tuned populations of visual neurons. Other studies directly probe the accuracy of motor uncertainty estimates employed in visuomotor tasks. They reveal misestimation of motor uncertainty, finding either under- or overestimation of uncertainty [19,20]. Uncertainty-based inference underpins many human behaviours, yet clear evidence that these inferences rely on accurate uncertainty estimates has been limited to a few visual features. This lack of behavioural evidence may be due to nontrivial methodological and computational challenges.

First, many parameters influence human behaviour similarly, making it challenging to attribute changes in behaviour to a single parameter. Specifically, it is difficult to isolate the estimate of sensory uncertainty from Bayesian priors and biases. Let’s continue with the dog-search example. Previous experience might tell you that your dog often dashes towards its favorite rabbit hole, left of where you heard the bark. Optimally, this knowledge would steer you to the left of the bark, with the exact deviation depending on the spatial uncertainty of the bark and the precision of your spatial representation of the rabbit hole. If you nevertheless sprint straight towards the bark, there are at least three explanations for your behaviour. (i) The bark provided precise spatial information, leading you to rely mostly on the auditory measurement. (ii) The bark provided uncertain spatial information, but your inference relied on an underestimate of that spatial uncertainty, again leading to dominance of the auditory measurement over prior knowledge. (iii) Your auditory spatial perception has a rightward bias; consequently, even though you integrated the rabbit hole prior, the final location estimate happened to align with the true location of the bark. Many studies conclude that the brain employs accurate estimates of sensory uncertainty based on good matches between human behaviour and predictions of a Bayesian ideal-observer model [4,21–24]. However, in these models, sensory uncertainty and priors trade off. Even if the prior is learned during the experiment, it might be learned imprecisely [25–27], and its final representation is inaccessible to the experimenter, as are the estimates of sensory uncertainty employed for perceptual inference.

**Figure 2.**
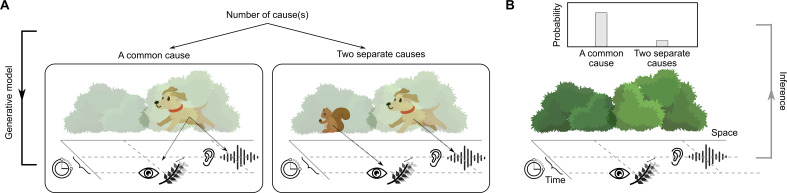
Role of causal inference in multisensory tasks. (A) A generative model describing the statistical structure of the physical world resulting in visual and auditory signals arriving in the participant’s brain. The two spatially and temporally discrepant measurements may originate from a single source (a barking dog whose shuffling visibly shakes branches of the bush), or two different sources (a squirrel shaking the bush and a barking dog). (B) Inference about whether there is one common or two separate sources based on the visual and auditory measurements.

Second, the model has to capture the complexity of inferences about the world that underlie observed behaviour. Continuing with the search for your dog, suppose in addition to the sound, you spot movement behind a nearby bush. Optimally combining the two pieces of information based on their relative precision will reduce spatial uncertainty [28–30]. You might aim more towards the shaking bush as vision usually provides more precise spatial information than audition [31–34]. Hence, multisensory integration provides a strong test case for uncertainty-based perceptual inference. Some studies report variance reduction predicted by optimal cue integration [21,28–30,35–40], while others fail to find this [41–48]. However, multisensory integration only makes sense when the visual and auditory information originate from the same event [49,50]. If the moving twigs are far from the apparent location of the dog’s bark or you saw the movement long after hearing the sound, then you would probably sprint straight towards the bark (figure 2A). Yet, you might also steer towards the bark because you underestimated its spatial uncertainty. Both plausible scenarios—doubts about the shared origin of the sensory signals and employing inaccurate estimates of sensory uncertainty—will lead to apparently suboptimal cue combination. Thus, models must infer the causal structure of the sensory signals [27,32,49,51–60] to draw conclusions about the estimates of sensory uncertainty underlying perceptual inference (figure 2B). Sensory biases affect causal inference [61] and priors over stimulus properties influence integration, stressing the need to account for potential trade-offs between model parameters when accounting for the complexity of the observer’s world model.

Here, we test whether accurate estimates of auditory temporal and spatial uncertainty underlie perception across unimodal and bimodal contexts. Participants completed a series of psychophysical experiments, encompassing temporal-order judgments, spatial discrimination and localization in unimodal and bimodal contexts as well as explicit causal-inference judgments. To address the challenge of trade-offs between parameters, we jointly fitted each participant’s data from all experiments, allowing us to constrain all parameters that could explain the observed behaviour. We contrasted models in which inaccurate estimates of temporal and/or spatial uncertainty were employed for perceptual inference with a model that assumed accurate uncertainty estimates. To foreshadow the results: participants typically relied on inaccurate estimates of auditory spatial uncertainty in both unimodal and bimodal contexts as well as on inaccurate estimates of audiovisual temporal uncertainty. Parameter estimates indicate that participants used underestimates of auditory temporal and spatial uncertainty for perceptual inference.

## 2. Methods

### (a) Participants

Thirteen participants (seven females, aged 21−34 years; 12 naive with respect to the purposes of the experiment; all right-handed) were recruited. One participant’s uncertainty estimates in the audiovisual context were excluded from one of the statistical tests, as the estimate was pinned at the generously set boundary. All of them stated having no visual, auditory or motor impairments. The experiment was approved by the institutional review board of New York University and all participants gave written informed consent prior to beginning the study.

### (b) Apparatus and stimuli

The experiment was conducted in a dark, semi-sound-attenuated room. Participants were seated 1 m from an acoustically transparent screen (1.36 × 1.02 m, 68 × 52 deg visual angle) with their head stabilized by a chin rest. Visual stimuli were high-contrast Gaussian blobs (s.d.: 3.6 deg) against a black background, projected onto the screen for 100 ms. Behind the screen, a loudspeaker was mounted on a computer-controlled sledge attached to a 1.5-metre-long linear rail, which was hung from the ceiling perpendicular to the line of sight. The auditory stimuli were 100 ms-long noise bursts (20 Hz–20.05 kHz, 60 dB) windowed using the positive half of a sine wave with a period of 200 ms. Participants’ dominant hand rested on a rollerball mouse with two buttons. Stimulus presentation, speaker movement, and data collection were controlled by an iMac running MATLAB R2017b. Visual and auditory stimuli were presented using the Psychophysics Toolbox [62].

### (c) Procedure

**Figure 3.**
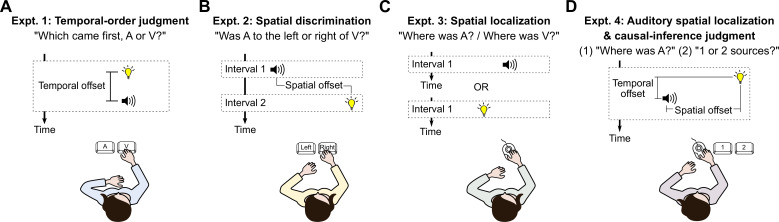
Experiments. (A) In Expt. 1, an audiovisual stimulus pair with variable temporal offset was presented at the central location. Participants reported which modality they perceived first. (B) In Expt. 2, a visual and an auditory stimulus were presented sequentially with variable spatial offset. Participants reported whether they perceived the auditory stimulus to the left or to the right of the visual stimulus. (C) In Expt. 3, either a visual or an auditory stimulus was presented at a random location. Participants indicated the perceived location by moving a cursor to the corresponding point on the screen. (D) In Expt. 4, an audiovisual stimulus pair with varying spatial and temporal offsets was presented. Participants first moved a cursor to the perceived horizontal location of the auditory stimulus and then reported by button press whether they perceived the auditory and the visual stimulus as sharing a common source or originating from two separate sources.

In Expt. 1, participants completed a two-alternative, forced-choice audiovisual temporal-order-judgment task (figure 3A; electronic supplementary material, S1.1). In each trial, a visual and an auditory stimulus were presented at the central location with varying SOA (20 levels ranging from −466.67 to 466.67 ms, negative values indicate visual-first stimulus pairs). Participants reported by button press which stimulus they had perceived first. Each SOA was presented 25 times, resulting in a total of 500 trials administered in pseudorandom order. Participants usually took an hour to complete this experiment.

In Expt. 2, participants completed a two-interval, forced-choice audiovisual spatial-discrimination task (figure 3B; electronic supplementary material, S1.2). In each trial, an auditory and a visual stimulus were presented in random order with an SOA of 1600 ms. Participants reported by button press whether they perceived the auditory stimulus to the left or right of the visual stimulus. The visual stimulus was presented at either −12 or 12 deg relative to the centre of the screen. The location of the auditory stimulus was controlled by four interleaved staircases, two for each visual stimulus location. Each staircase comprised 40 trials, resulting in a total of 160 trials. Participants usually took about an hour to complete this experiment.

In Expt. 3, participants completed an auditory and a visual spatial-localization task (figure 3C; electronic supplementary material, S1.3). In each trial, either an auditory or a visual stimulus was presented. Participants moved a visual cursor to the stimulus location. The visual stimulus was presented at either ±4 or ±12 deg relative to the centre of the screen. Auditory stimulus locations were those for which each participant perceived auditory stimuli as aligned with visual stimuli presented at ±12 deg in Expt. 2. Each visual stimulus location was tested 20 times, and each auditory location was tested 40 times, resulting in 160 trials administered in pseudorandom order. Participants took an hour and a half to complete the experiment.

In Expt. 4, participants completed two tasks, an auditory spatial-localization task and a two-alternative, forced-choice causal-inference task (figure 3D; electronic supplementary material, S1.4). In each trial, an audiovisual stimulus pair was presented. Participants first moved a visual cursor to the horizontal position of the auditory stimulus, and then reported by button press whether the two stimuli originated from the same source or from separate sources. The visual stimulus was presented at ±4 or ±12°; the auditory stimulus was presented at one of the same two locations as in Expt. 3, those at which the auditory stimuli were perceptually aligned with visual stimuli at ±12 deg. The stimuli were presented with an SOA of 0, ±250, or ±400 ms (negative values indicate visual first). There were 40 different audiovisual stimulus pairs (4 visual stimulus locations × 2 auditory stimulus locations × 5 SOAs), each of which was tested 20 times, resulting in 800 trials. Participants usually took 3 h to complete the entire experiment.

Response feedback was never provided. The four experiments were split across three sessions. During the first session, participants completed Expts. 2 and 3; in the second session, they completed about 65% of Expt. 4; in the third session, they completed the rest of Expt. 4, followed by Expt. 1. For further experimental details, see electronic supplementary material, S1.

## 3. Modelling

We begin with the general assumptions of the models and then briefly describe the model variants we tested and how they fit to the data. A comprehensive specification of the models and their parameters is given in electronic supplementary material, S5.

### (a) General model assumptions

First, each feature of a stimulus in the world s is represented by a sensory measurement $m^{\prime }$ in a participant’s brain. Based on previous work [27,63,64], we assumed the auditory measurements might be biased. Thus, the average auditory measurement equals a remapped feature value $s^{\prime }$, which is a linear function of the physical feature value s. More specifically, $s_{A}^{\prime }=a_{A}s_{A}+b_{A}$, where $a_{A}$ represents a location-dependent bias term and $b_{A}$ a location-independent bias term. On the other hand, we assumed the average visual measurement equals the physical feature value, that is, $s_{V}^{\prime }=s_{V}$.

Second, the measurements are corrupted by Gaussian noise $m^{\prime }∼N(s^{\prime },{\sigma^{\prime }}^{2})$. The standard deviation of repeated measurements across trials reflects the actual sensory uncertainty $\sigma^{\prime }$ associated with the stimulus. We assume, based on pilot data (electronic supplementary material, S8.1), that the uncertainty is constant across stimulus locations (figure 1B).

Third, the participant has prior knowledge (or prior assumptions) about the probability distribution of the stimulus features. For example, the participant might assume that stimuli are most likely located straight ahead, $s∼N(\mu_{p}=0,{\sigma_{P}^{\prime }}^{2})$ [49].

Fourth, in every trial, the participant derives the posterior probability of a stimulus feature by combining the prior and the likelihood. Because the sensory noise is Gaussian, the likelihood is a Gaussian function centred on the measurement. The standard deviation of the likelihood corresponds to the estimated uncertainty, ${\tilde{\sigma }}^{\prime }$, which might differ from the actual sensory uncertainty, ${\tilde{\sigma }}^{\prime }=\sigma^{\prime }$ or ${\tilde{\sigma }}^{\prime }\neq \sigma^{\prime }$ (figure 1C). We test for differences between the actual and the employed sensory uncertainty for audition, that is, for the uncertainty associated with auditory spatial-location measurements ($\sigma_{A}^{\prime }$ vs. ${\tilde{\sigma }}_{A}^{\prime }$) and audiovisual SOA measurements ($\sigma_{\Delta_{t}}^{\prime }$ vs. ${\tilde{\sigma }}_{\Delta_{t}}^{\prime }$).

Fifth, the participant uses the mode of the posterior as the (conditional) estimate of the stimulus feature.

Sixth, the task-relevant estimates of the stimulus feature(s) are used to generate a perceptual decision. Depending on the task, perceptual decisions might be based directly on one or two feature estimates or might involve further inferences such as those over the causal scenario underlying auditory and visual measurements.

Finally, the response specifying the perceptual decision is subject to additional noise sources such as motor noise and attentional lapses.

### (b) Model variants

We tested four models: Either none, one, or both of the uncertainty estimates, ${\tilde{\sigma }}_{\Delta_{t}}^{\prime }$ or ${\tilde{\sigma }}_{A}^{\prime }$, were independent of the actual uncertainty $\sigma_{\Delta_{t}}^{\prime }$ or $\sigma_{A}^{\prime }$. We fitted each model jointly to the data from all four experiments. For example, if in a model perceptual inferences were based on an accurate estimate of auditory spatial uncertainty (${\tilde{\sigma }}_{A}^{\prime }=\sigma_{A}^{\prime }$), this held for the modelling of all inferences that used an estimate of auditory spatial uncertainty. Specifically, Expt. 1 constrained $\sigma_{\Delta_{t}}^{\prime }$, Expts. 2 and 3 constrained $\sigma_{A}^{\prime }$ and ${\tilde{\sigma }}_{A}^{\prime }$, and Expt. 4 constrained $\sigma_{AV,A}^{\prime }$, ${\tilde{\sigma }}_{AV,A}^{\prime }$, $\sigma_{\Delta_{t}}^{\prime }$ and ${\tilde{\sigma }}_{\Delta_{t}}^{\prime }$. Based on previous results [55,65], we assumed that auditory spatial uncertainty might differ in unimodal (Expts. 2 and 3) compared to bimodal contexts (Expt. 4, so that $\sigma_{AV,A}^{\prime }\neq \sigma_{A}^{\prime }$).

### (c) Model fitting

The models of the five tasks were fitted jointly; the log-likelihood of the full parameter set (see electronic supplementary material, tables S4–S6) was calculated by combining the log-likelihoods of each model and its parameters. The best-fitting parameters, i.e. the parameters for which the joint likelihood was maximal, were derived using the BADS toolbox [66]. This joint fit was repeated for each of the four model variants. To compare model performance across model variants, we computed the Akaike information criterion (AIC) [67] separately for each participant. The winning model corresponded to the model with the lowest AIC value. We compared the best-fitting parameter values of the actual to the employed uncertainty using pairwise t-tests, separately for audiovisual temporal and auditory spatial uncertainty.

## 4. Results

To identify whether participants employed correct estimates of their temporal and spatial sensory uncertainty for perceptual inference, we contrasted a model that assumed participants’ perceptual decisions relied on accurate estimates of their audiovisual temporal and auditory spatial uncertainty with models that allowed for inaccurate estimates of uncertainty. We further distinguished models that assumed decisions based on inaccurate temporal uncertainty, inaccurate spatial uncertainty, or both.

Each model was jointly fitted to a participant’s responses in five tasks tested in four separate experiments: (i) audiovisual temporal-order judgment, (ii) audiovisual spatial discrimination, (iii) auditory and visual spatial-localization, (iv) auditory spatial-localization for auditory stimuli accompanied by a temporally and/or spatially discrepant visual stimulus, and (v) a causal-inference task in which participants determined the number of sources of these audiovisual stimulus pairs (see figure 3).

**Figure 4.**
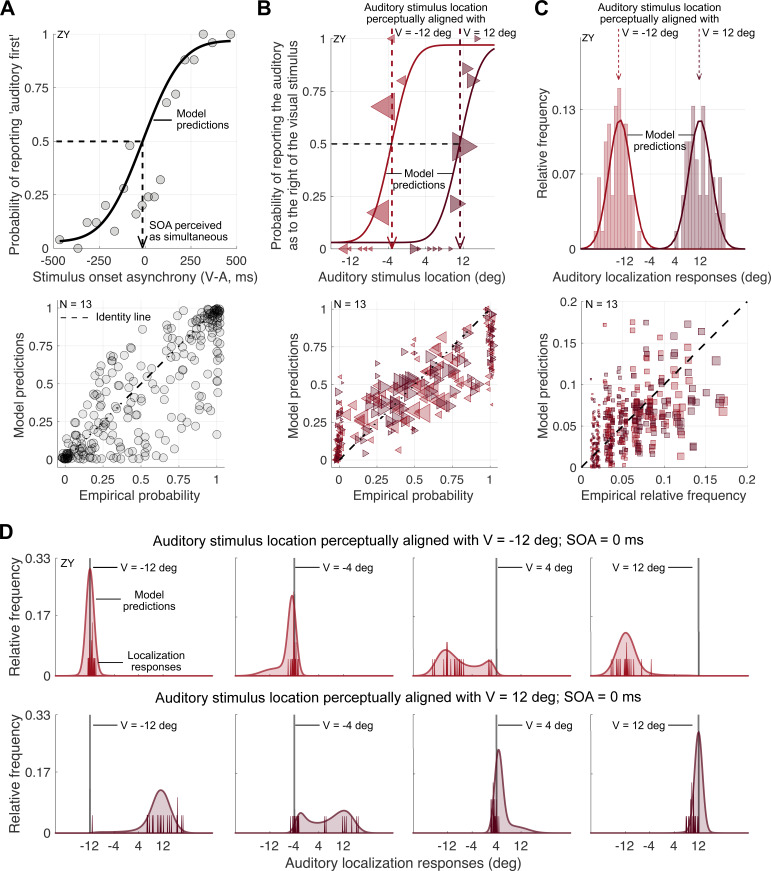
Behavioural results and joint model predictions for audiovisual temporal and spatial discrimination tasks as well as auditory localization in unimodal and bimodal contexts. (A) Expt. 1 (audiovisual temporal-order judgment). Top panel: observed (grey dots) and predicted (solid line) proportions of auditory-came-first reports as a function of stimulus onset asynchrony (SOA) of the auditory and visual stimulus (negative values: visual first) for an example participant. Bottom panel: probability of reporting auditory-came-first, empirical data versus model predictions for all participants and SOAs. (B) Expt. 2 (audiovisual spatial discrimination). Top panel: observed proportion of trials in which the auditory stimulus was reported as located to the right of the visual one, plotted as a function of the binned auditory stimulus location (triangular markers, area proportional to the number of trials) for the same participant as in (A). Observed proportions and model predictions (lines) are shown separately for trials in which the visual stimulus was presented at −12 deg (leftward-pointing light red triangles) and trials in which the visual stimulus was presented at 12 deg (rightward-pointing dark red triangles). Dashed arrows: auditory stimulus locations perceptually aligned with visual stimuli at −12 and 12 deg. These participant-specific auditory stimulus locations were used in all other experiments. Bottom panel: probability of reporting the auditory stimulus as located to the right of the visual one, (binned) empirical data versus model predictions for all participants. Marker size is proportional to the number of trials per bin. (C) Expt. 3 (auditory localization in unimodal contexts). Top panel: histogram of localization responses and model predictions (lines) for the same participant. The auditory stimulus was presented at the location perceptually aligned with a visual stimulus at −12 deg (light red) or to a visual stimulus at 12 deg (dark red). Auditory and visual stimuli were interleaved (see electronic supplementary material, S3 for data from the visual-localization task). Bottom panel: relative frequencies of observed versus predicted binned localization responses for all participants. (D) Expt. 4A (auditory localization in audiovisual contexts). Observed (solid vertical red lines) and predicted (shaded area) frequencies of localization responses for auditory stimuli presented as part of an audiovisual stimulus pair with a SOA of 0 ms for the same participant. The auditory stimulus was either presented at a location perceptually aligned with a visual stimulus at −12 deg (top row) or 12 deg (bottom row). The visual stimulus was presented at one of four locations (±4, 12 deg; columns).

**Figure 5.**
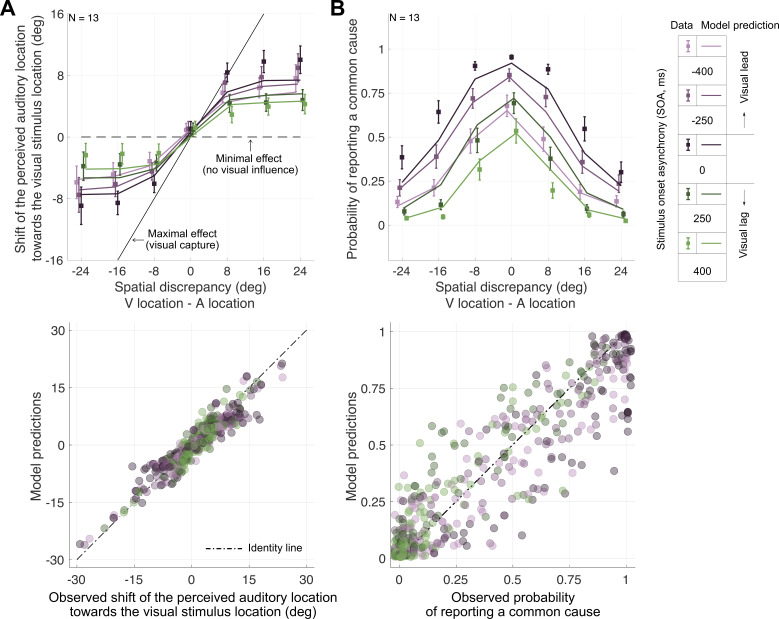
Behavioural results and model predictions for auditory localization in bimodal contexts and audiovisual causal-inference judgments. (A) Top panel: Group average shifts of perceived auditory location towards the visual stimulus location for each audiovisual temporal discrepancy as a function of audiovisual spatial discrepancy. See electronic supplementary material, figure S10 for a depiction of the perceptual shift conditioned on the response in the explicit causal-inference task. Data are shifted slightly horizontally for legibility. Bottom panel: observed versus predicted shifts in auditory localization responses towards the visual location. (B) Probability of reporting a common cause as a function of spatial discrepancy for each temporal discrepancy (colours). Markers: group averages, error bars: ±1 SEM. Bottom panel: observed versus predicted proportion of common-cause responses.

The behavioural results in all experiments showed clear signatures of task-specific perceptual inference, known to rely on estimates of the uncertainty associated with a sensory measurement (see figure 4 for the data of a representative participant and electronic supplementary material, S3 for all participants). Responses in the audiovisual spatial-discrimination task (Expt. 2) showed biases (figure 4B; electronic supplementary material, S2 and S3.2) that were also reflected in the localization of auditory (figure 4C) but not visual (electronic supplementary material, S3.3) stimuli in the unimodal localization task (Expt. 3). In the bimodal context of Expt. 4, auditory localization responses were shifted towards the accompanying visual stimulus, indicative of multisensory integration (figure 4D; electronic supplementary material, S3.4). Moreover, these perceptual shifts as well as the proportion of trials in which participants perceived the auditory and the visual stimulus as sharing a common cause decreased with increasing spatial and temporal discrepancy, demonstrating that participants performed causal inference (figure 5A,B top panels; see electronic supplementary material, S3.4 for statistical analysis).

We assumed that parameters that influenced perceptual decisions did so consistently across all five tasks. Thus, we modeled data from these five tasks jointly, assuming consistent parameter values across tasks. Jointly fitting a set of observer models to all of the data allowed us to simultaneously constrain parameters of interest, those reflecting the participant’s actual and employed temporal and spatial auditory uncertainty, as well as additional parameters that might trade off against the parameters of interest, including biases in auditory spatial perception, biases in audiovisual temporal perception, and prior assumptions regarding the prevalence of some spatial locations and temporal discrepancies. The validity of this approach is demonstrated in the general agreement between data and model predictions across all tasks (figure 4A-C, bottom panels).

The behavioural results were best captured by a model variant that allowed perceptual inferences to be based on inaccurate estimates of both temporal and spatial uncertainty (11 out of 13 participants, see electronic supplementary material, S6 for measures of model fit for each model variant and participant and for model comparisons). The behaviour of the two remaining participants was best captured by model variants that assumed that only inaccurate temporal or inaccurate spatial uncertainty estimates were employed.

**Figure 6.**
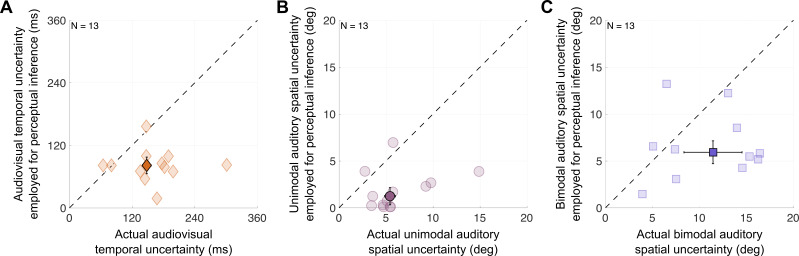
Systematic deviance between auditory spatial and audiovisual temporal uncertainty and the estimates used for perceptual inference. Participants' actual sensory uncertainties (the standard deviations of the measurement distributions) are plotted against the uncertainty values they used for perceptual inference (the standard deviations of the likelihood functions). (A) Audiovisual temporal uncertainty. (B) Auditory spatial uncertainty given an auditory stimulus presented alone or (C) accompanied by a visual stimulus. Darker markers: group medians. Error bars: ±SEM. Dashed lines: identity. One outlier is not displayed in (C) to avoid excessive scaling of the axes (see electronic supplementary material, S6). All shown values are the best-fitting parameter values of the winning model.

Parameter estimates of the winning model revealed how gravely and consistently participants underestimated their sensory uncertainty, that is, by how much the uncertainty estimates participants used for perceptual decisions differed from their actual sensory uncertainty. The temporal uncertainty estimates participants’ employed for inference (median = 74.93 ± 14.02 ms) were about half of their actual temporal uncertainty (median = 157.68 ± 14.02 ms; t(12)=4.3, p=0.001; figure 5A). The same mismatch was observed for auditory spatial uncertainty in unimodal and bimodal contexts: the employed uncertainty was less than half of the actual uncertainty (figure 6B,C; unimodal context: median = 1.46 ± 0.41 deg vs. 5.45 ± 0.92 deg; t(12)=4.79, p<0.001; bimodal context: median = 7.18 ± 4.91 deg vs. 15.58 ± 1.93 deg; t(11)=2.79, p=0.018). These results provide clear evidence that humans’ inferences rely on underestimates of audiovisual temporal and auditory spatial uncertainty.

## 5. Discussion

In this study, we tested the ubiquitous assumption that perceptual inference relies on accurate estimates of sensory uncertainty. Participants completed a series of experiments encompassing audiovisual temporal-order judgments, audiovisual spatial discrimination, auditory spatial localization in unimodal and bimodal contexts, as well as causal-inference judgments. Perceptual decisions in these experiments depend on the participant’s auditory and visual spatial uncertainty as well as their audiovisual temporal uncertainty. To evaluate whether participants’ perceptual inferences relied on accurate estimates of these sensory uncertainties, we contrasted models that differed in the agreement between actual and employed temporal and spatial uncertainty. Data from all tasks were fitted jointly to constrain estimates of all components of perceptual inference. Model comparisons and evaluation of the best-fitting parameters revealed that the majority of participants systematically based their perceptual inferences on underestimates of audiovisual temporal and auditory spatial uncertainty. This pattern is held across unimodal and bimodal contexts, indicating that the revealed mismatch between actual and employed sensory uncertainty is a general phenomenon.

The results revealed that participants used underestimates of auditory spatial and audiovisual temporal uncertainty for various perceptual inferences. In contrast, visual-spatial uncertainty—consistent with previous studies [25]—was assumed to be accurately represented, suggesting that the quality of uncertainty estimates varies across modalities. This discrepancy might be rooted in fundamental differences between the encoding of spatial location in primary visual and auditory cortices. Bayesian theories of neural coding propose that sensory uncertainty is automatically encoded in the population response of neurons that are tuned to the feature of interest [11]. In the visual system, various features such as location, orientation, and motion direction are encoded by tuned populations [10,68,69], rendering such a mechanism possible. In contrast, spatial location in the auditory cortex is coded by opponent neural populations that are broadly tuned for far-left or far-right locations [70,71]. Thus, models that describe the neural representation of uncertainty for visual features cannot be easily extended to auditory spatial uncertainty.

It seems unlikely that a probabilistic population code is used for temporal discrepancy as there is no specific sensory system specialized for time perception. Neuroimaging research suggests that temporal-order information is processed in a distributed network of brain regions across prefrontal, fronto-parietal, parietal, and occipito-temporal cortices [72–74]. Given that multiple brain areas are involved in encoding cross-modal temporal discrepancy, the representation of sensory uncertainty might be similarly distributed and difficult to decode. Estimation of temporal uncertainty might require combining information across these regions, which could lead to a mismatch between actual and employed temporal uncertainty.

We observed a consistent use of underestimated sensory uncertainty across participants, tasks and domains, which puts the costs and benefits of such underestimates into focus. Simulation results suggest that basing perceptual decisions on underestimated sensory uncertainty improves decision accuracy at the expense of precision. However, the tradeoff between accuracy and precision appears to be beneficial only when the internally remapped stimulus location closely matches the physical stimulus location (electronic supplementary material, S4). The presence of biases in auditory spatial perception undermines the possibility of achieving accuracy through underestimated sensory uncertainty. Nonetheless, perceptual biases are inherent and likely to remain undetected by individuals [63].

The brain constantly makes decisions based on noisy sensory information. The extent to which the brain employs an accurate estimate of sensory uncertainty for these decisions has profound implications for perception, action and cognition. Sensory uncertainty is a critical variable in a myriad of models describing human behaviour across a wide range of domains, from perceptual inference [75,76] to cognitive and metacognitive judgments [77–79] to sensorimotor control [80]. Central to these models is the assumption that inference relies on accurate estimates of sensory uncertainty. Our findings suggest a critical revision: models should include the possibility that sensory uncertainty is not accurately represented. The spread of the likelihood function that underlies an inference might not match that of the distribution of noisy measurements across trials. Incorporating this mismatch introduces greater model complexity, increasing the risk of overfitting by adding more degrees of freedom. To mitigate this risk, researchers might emphasize experimental designs ensuring that all parameters are properly constrained.

To further support our findings, we explored whether our modelling choices could account for the observed data. More specifically, we independently tested a model-selection approach [32] for deriving auditory spatial estimates in the bimodal context, and a posterior-probability-based rule for determining the explicit causal-inference judgments. Even when these decision strategies were combined with the use of mismatched uncertainties, the jointly fitted model performed significantly worse than the winning model (electronic supplementary material, S7). Thus, suboptimal decision-making alone cannot explain the observed data. Notably, the combination of optimal causal-inference-based spatial estimation and suboptimal explicit causal inference used here also emerged from extensive model comparisons in our previous visual-tactile [55] and visual-auditory studies [27].

Our models further assume that the measurement distributions are symmetric Gaussian distributions with a fixed standard deviation, independent of spatial location. For visual perception, it is well established that uncertainty increases with eccentricity [63]. Such location-dependent uncertainty could result in asymmetric likelihood functions [24]. However, because our experiments did not control visual fixation, incorporating spatially dependent visual uncertainty would introduce unjustified complexity. In contrast, head position was fixed throughout the experiments, potentially giving rise to variations in auditory spatial uncertainty across horizontal positions. However, auditory localization data suggest no systematic variation of auditory uncertainty with stimulus location in our setup (see electronic supplementary material, S8.1). Similarly, stimulus uncertainty might increase with time after the stimulus presentation, potentially resulting in order effects. Our data did not show a systematic order effect in the 2IFC audiovisual spatial-discrimination task (see electronic supplementary material, S8.2). Although not observed in our data, these factors could influence uncertainty and with it the likelihood functions, raising the question of whether perceptual inferences account for these types of systematic changes in uncertainty.

In conclusion, this study revealed that perceptual inference across different contexts relies on underestimates of auditory spatial and audiovisual temporal sensory uncertainty.

## Data Availability

All data and code are publicly available on the Open Science Framework [81]. Supplementary material is available online [82].
